# Enhancing the scalability of *Wolbachia*-based vector-borne disease management: time and temperature limits for storage and transport of *Wolbachia*-infected *Aedes aegypti* eggs for field releases

**DOI:** 10.1186/s13071-023-05724-1

**Published:** 2023-03-18

**Authors:** Megan J. Allman, Ya-Hsun Lin, D. Albert Joubert, Jessica Addley-Cook, Maria Camila Mejía-Torres, Cameron P. Simmons, Heather A. Flores, Johanna E. Fraser

**Affiliations:** 1 Institute of Vector-borne Diseases, Monash University, Melbourne, VIC 3800 Australia; 2 Department of Microbiology, Monash University, Melbourne, VIC 3800 Australia; 3 World Mosquito Program, Monash University, Melbourne, VIC 3800 Australia; 4 School of Biological Sciences, Monash University, Melbourne, VIC 3800 Australia

**Keywords:** *Wolbachia*, *Aedes aegypti*, Insect releases, Capsule, Egg release, Endosymbiont, Biocontrol

## Abstract

**Background:**

Introgression of the bacterial endosymbiont *Wolbachia* into *Aedes* *aegypti* populations is a biocontrol approach being used to reduce arbovirus transmission. This requires mass release of *Wolbachia*-infected mosquitoes. While releases have been conducted using a variety of techniques, egg releases, using water-soluble capsules containing mosquito eggs and larval food, offer an attractive method due to its potential to reduce onsite resource requirements. However, optimisation of this approach is required to ensure there is no detrimental impact on mosquito fitness and to promote successful *Wolbachia* introgression.

**Methods:**

We determined the impact of storage time and temperature on wild-type (WT) and *Wolbachia*-infected (*w*Mel or *w*AlbB strains) *Ae. aegypti* eggs. Eggs were stored inside capsules over 8 weeks at 18 °C or 22 °C and hatch rate, emergence rate and *Wolbachia* density were determined. We next examined egg quality and *Wolbachia* density after exposing eggs to 4–40 °C to determine how eggs may be impacted if exposed to extreme temperatures during shipment.

**Results:**

Encapsulating eggs for 8 weeks did not negatively impact egg viability or resulting adult emergence and *Wolbachia* density compared to controls. When eggs were exposed to temperatures within 4–36 °C for 48 h, their viability and resulting adult *Wolbachia* density were maintained; however, both were significantly reduced when exposed to 40 °C.

**Conclusions:**

We describe the time and temperature limits for maintaining viability of *Wolbachia*-infected *Ae. aegypti* eggs when encapsulated or exposed to extreme temperatures. These findings could improve the efficiency of mass releases by providing transport and storage constraints to ensure only high-quality material is utilised during field releases.

**Graphical Abstract:**

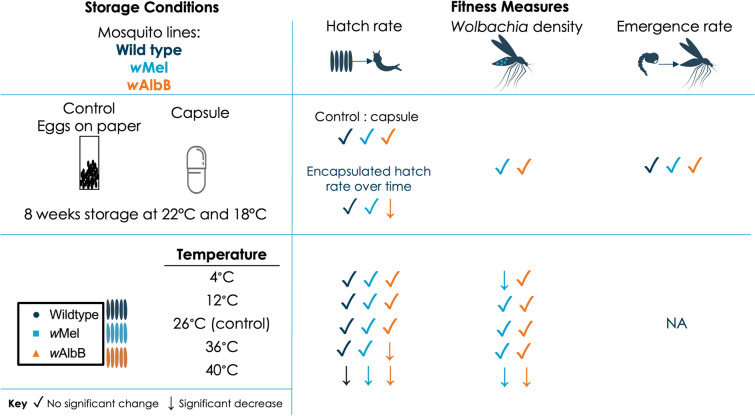

**Supplementary Information:**

The online version contains supplementary material available at 10.1186/s13071-023-05724-1.

## Background

Dengue, caused by dengue virus (DENV), is endemic in over 100 countries, with approximately half of the world’s population at risk of infection [1–3]. *Aedes aegypti* is the major vector for DENV as well as Zika virus (ZIKV), chikungunya virus (CHIKV) and yellow fever virus [4, 5]. The prevalence of these viral diseases is continuing to rise because of vector habitat expansion [6, 7] and failure of current prevention strategies such as insecticides [8].

This has consequently driven the development of several novel biocontrol strategies over the last decade. Population suppression methods involve the release of male insects sterilised by chemical exposure, irradiation or genetic modification [9–12]. Sterile males then mate with wild females to produce inviable offspring, reducing the population size. Another way to prevent females producing offspring is by releasing incompatible males. This method has been developed through the introduction of the endosymbiotic bacterium *Wolbachia* into *Ae. aegypti*. When *Ae. aegypti* are transinfected with *Wolbachia*, male sperm are reproductively modified such that when males mate with wild females, their offspring die [13–16]. All current population suppression technologies involve the continual release of males which reduce the population over time. Alternatively, *Wolbachia* can be used in a population introgression approach. When *Ae. aegypti* mosquitoes carry *Wolbachia*, the transmission potential for viruses such as DENV [14, 17, 18], ZIKV [19, 20], CHIKV [14, 19, 21] and yellow fever virus [21, 22] is reduced. This approach involves the release of both male and female *Wolbachia*-infected *Ae. aegypti.* While *Wolbachia*-uninfected females do not produce viable eggs when mated with *Wolbachia*-infected males, *Wolbachia*-infected females can rescue this lethality, providing them a reproductive advantage. *Wolbachia* is maternally inherited such that, over time, *Wolbachia* spreads through the population, creating a mosquito population that is refractory to viral transmission. All of these biocontrol methods are dependent on the mass release of mosquitoes that are competitive with natural populations. Therefore, having the tools to implement biocontrol methods that offer a long-term solution, at a scale sufficient to address the significant distribution of mosquito-borne viruses, is of high priority [23–27].

To introduce mosquitoes into the wild, eggs, pupae or adult releases have been used. Pupae release devices hold pupae in water and provide protection and sucrose for emerged adults. Release at the pupae stage of life is beneficial because, unlike larvae, pupae do not require food [28, 29]. However, to achieve synchronised development en masse is very difficult as the pupal stage only lasts for approximately 24 h. Adult releases typically involve the packaging of adults in ventilated plastic tubes and manually releasing them from either a slow-moving (30–35 km/h) vehicle or on foot [30]. Aerial release of adults has been investigated in the context of the sterile insect technique, and it involves a specialised release mechanism that stores up to 50,000 mosquitoes, maintains cool temperatures and doses and ejects mosquitoes when prompted [31–33]. Aerial releases would greatly reduce operational costs; however, they are not practical in all geographical and social contexts, such as certain climate conditions and informal settlements, meaning ground releases are still of importance. Given that ground release of pupae and adults requires substantial resources to rear and package mosquitoes, egg releases offer an attractive alternative. Release of *Wolbachia*-infected *Ae. aegypti* eggs involves the production of eggs at an onsite facility or at a regional hub and shipment to sites where eggs are released into the field in a container of water with sufficient larval food. Egg releases are applicable to any biocontrol methods that do not require sex sorting prior to field release, such as Oxitec’s Friendly™ capsule method, gene drive and other genetically modified mosquito releases [34, 35]. This method has successfully been used to establish *Wolbachia* in *Ae. aegypti* populations the field, for example in parts of Queensland, Australia, and Yogyakarta, Indonesia [36–38]. However, aliquoting and distributing eggs with larval food while maintaining egg viability is difficult en masse; hence, to improve the egg packaging and delivery method, encapsulation of eggs with larval food in water-soluble capsules has been developed [39].

Encapsulating eggs with larval food and hatching them with no storage time do not impact hatch rate, emergence rate, adult wing length or *Wolbachia* density [39]. However, the impact of extended storage time and temperature on encapsulated eggs remains unknown. Studies have shown that when *Wolbachia*-infected non-encapsulated eggs are stored over extended periods of time, egg viability decreases faster than in *Wolbachia*-free eggs [40–48]. Additionally, both low (< 14 °C) and high (cyclical 22–30 °C) egg storage temperatures have been shown to negatively impact egg viability [48, 49]. Therefore, it is important to determine whether encapsulation further exacerbates this impact and at what temperatures *Wolbachia*-infected eggs remain viable.

In this study, we investigate whether storage time or temperature impacts fitness measures of encapsulated eggs and whether *Wolbachia*-infected eggs (*w*Mel and *w*AlbB—the current strains being utilised in field releases [23, 50]) are impacted differently compared to WT eggs. We then examine the impact of exposure to extreme high and low temperatures on egg viability and *Wolbachia* density to inform appropriate egg transport and risk management strategies. We report that storing eggs inside of capsules does not negatively impact egg viability, emergence rates or *Wolbachia* density compared to the control storage method. We show that while egg viability is maintained quite well following exposure to cold temperatures, temperatures > 40 °C can reduce egg viability and *Wolbachia* density.

## Methods

### Mosquito strains and maintenance

Three Australian mosquito strains were used throughout this study: WT, *w*Mel- and *w*AlbB-infected *Ae. aegypti*. The establishment of these colonies has been previously described by Flores et al. [51]. Prior to the start of these experiments, the *w*Mel and *w*AlbB lines were backcrossed to Australian WT mosquitoes (100% WT males) for an additional three generations to reduce any genetic divergence that may have occurred between the strains. Additionally, partial backcrossing occurred each subsequent generation with 10% of WT males each generation. Experiments took place in the immediate one-eight generations after the completion of full backcrossing. Colonies were maintained under standard laboratory conditions in a climate-controlled insectary at 26 °C, 70% relative humidity (RH) with 12 h:12 h light/dark cycle.

### Larval diet

Larval diet was prepared by thoroughly grinding and mixing 35% beef liver powder (Now Foods, USA), 50% tuna meal (Ridley Aqua Feeds, Australia) and 15% brewer’s yeast (Now Foods, USA) together as described by Puggioli et al. [52]. The liquid diet version was prepared by mixing solid components with reverse osmosis (RO) water to form a 7.51% slurry. Food components were stored at 4 °C. In standard rearing conditions to generate eggs for experiments, shrimp wafers (Tetra®, USA) were used and stored at room temperature.

### Mosquito rearing

For each experiment, mosquitoes were reared for one generation to collect fresh eggs. To do this, eggs were vacuum hatched, and 200 larvae were placed in 3 l RO water and fed daily with liquid larval diet or shrimp wafers. At > 50% pupation, each container was transferred to a 24.5 × 24.5 × 24.5-cm or 20 × 20 × 30-cm cage and adult mosquitoes were provided with a sucrose solution (10% sucrose, 0.4% propionic acid). A blood meal was offered to adult females 5–6 days post emergence via artificial feeders. Human blood was provided by the Australian Red Cross (Supply Agreement 22-05VI-04) or human volunteers (Monash University Human Research Ethics permit 27690). Cups lined with filter paper and half filled with water were provided for oviposition; 96 h after blood feeding, paper substrates with mosquito eggs were dried by pressing between layers of paper towel and cloth for 2 h and then slowly dried over the course of the following day in shallow, paper towel-lined trays and stored at 26 °C and 75 ± 5% RH.

### Capsule production

To prepare egg capsules, 150 viable eggs were manually counted and gently brushed from the paper substrate into a size 00 water-soluble hydroxypropyl methylcellulose (HPMC) capsule (The Capsule Guy, Australia) using a small paintbrush. Prior to capsule preparation, hatch rate tests were conducted on eggs from all mosquito lines so that the number of viable eggs per capsule was accurately quantified. Capsules were then topped with 285 mg larval food and 110 mg activated charcoal. Activated charcoal was used as a filler to ensure capsules were completely full. For each experiment, five replicate capsules were prepared for each condition and hatch week. Food-only capsules were prepared and used as larval food for non-encapsulated control groups (i.e. eggs on paper substrate). Control group eggs were prepared by cutting the paper substrate that eggs were laid on into groups of approximately 150 viable eggs.

### Egg storage

For all storage conditions, eggs were maintained at 75 ± 5% RH and 22 °C unless specified otherwise. In temperature experiments, eggs contained in capsules or on paper substrate were stored at 18 °C or 22 °C. For extreme temperature experiments, eggs on paper substrate (unencapsulated) were stored at 4 °C, 12 °C, 26 °C, 36 °C or 40 °C. Temperature and humidity were controlled by storing eggs inside a laboratory incubator (Thermoline L + M) with a saturated salt solution and were tracked using hygrochrons (iButton®).

### Hatch rate

To determine the hatch rate of eggs in capsules or on paper substrate with a food capsule, 150–200 eggs per group were photographed and quantified, using Adobe Photoshop count tool, and submerged in cups with 300 ml RO water. The number of larvae in an individual container was counted 48 h after egg submersion. Larvae were returned to their corresponding containers after counting and allowed to develop to adulthood. Hatch rate was calculated as the percentage of eggs that produced larvae per container.

### Emergence rate

Emergence rate was determined 14 and 16 days post-hatching and was calculated as the percentage of larvae that emerged as adults per container.

### Wolbachia density

Six days post-emergence, adult females were collected (24–40 females per group) and placed individually into 96-well plates and homogenised in 50 μl squash buffer (10 mM Tris, pH 8.2; 1 mM EDTA; 50 mM NaCl) supplemented with 25 μg/ml proteinase K (Bioline) and a 2-mm glass bead (Pacific Laboratory Products). Samples were clarified by centrifuging for 3 min at 3000 rpm and then incubated in a thermocycler (5 min at 56 °C followed by 5 min at 98 °C). Mosquito homogenates were clarified again by centrifuging at 3000 rpm for 5 min and then supernatants diluted ten-fold using AE buffer (Qiagen). Total relative *Wolbachia* density was estimated by triplex quantitative polymerase chain reaction (qPCR). qPCR reactions were performed in 10 μl total volume containing 5 μl of 2 × LightCycler 480 Probes Master reaction mix, 2.5 μM primers, 10 μM of each probe (*Wolbachia surface protein [wsp], Ribosomal protein S17 [RpS17]* and the ankyrin repeat domain-containing protein (DEJ70_01140) in *w*AlbB [*wAlbB141*]) and 3 μl diluted (1:10) adult homogenate (see Additional file 1: Table S1 for probe and primer sequences) [53, 54]. Cycling was performed using LightCycler 480 II (Roche) with one cycle at 95 °C for 5 min, followed by 45 amplification cycles of 95 °C for 10 s, 60 °C for 15 s and 72 °C for 1 s. To analyse qPCR data Normalised Expressions (NE) were calculated using the delta Ct method [55], NE = 2^Cq (reference)^/2^Cq (target)^, where *RpS17* was the reference gene and *wsp* or *wAlbB141* the target gene.

### Statistical analysis

**Fig. 1:**
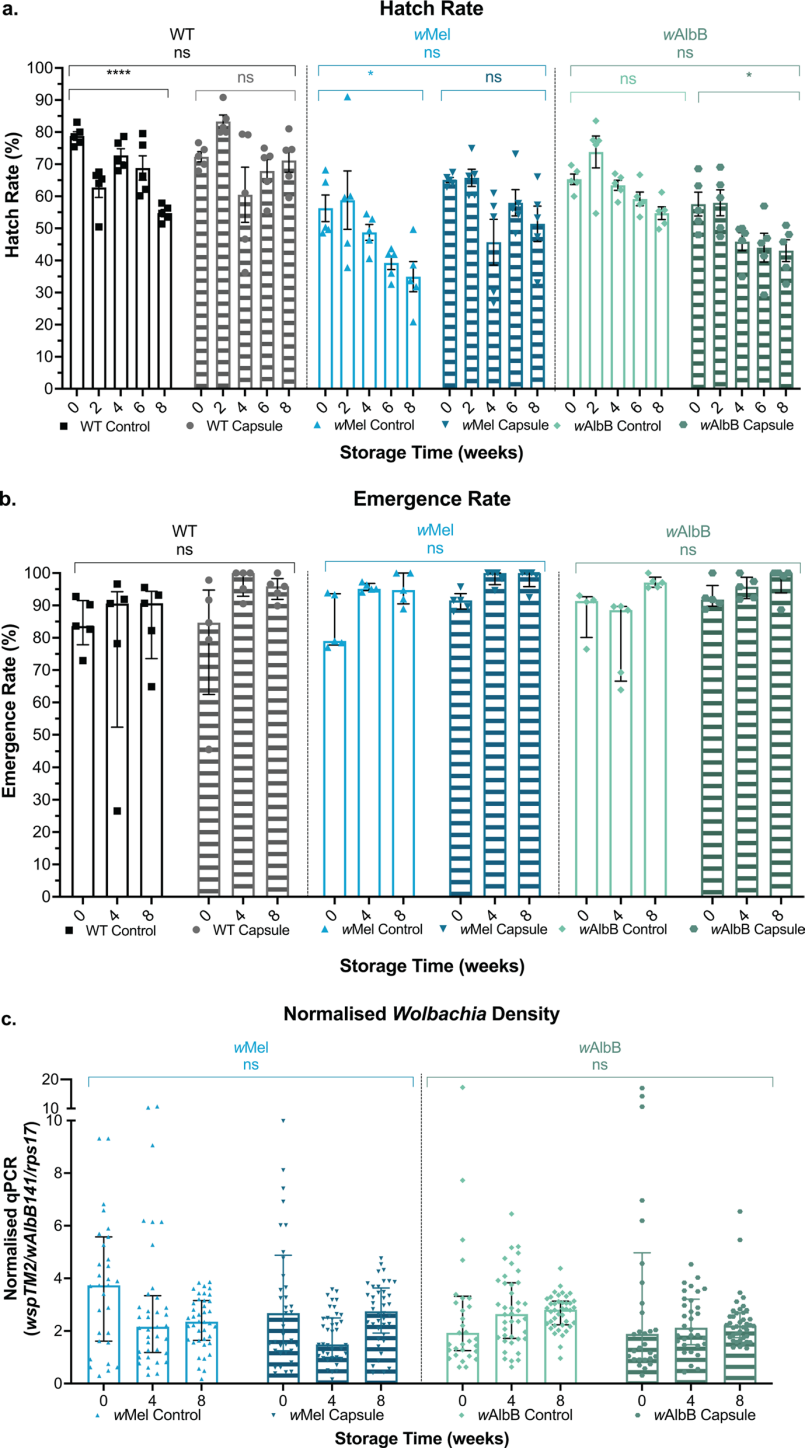
Encapsulating eggs for storage at 22 °C does not exacerbate impacts on egg viability, adult emergence or *Wolbachia* density compared to controls. WT, *w*Mel- and *w*AlbB-infected eggs were packaged into water-soluble capsules with larval food or left on paper substrate as a control and stored at 22 °C for 0, 2, 4, 6 or 8 weeks. **a** Hatch rate, **b** emergence rate and **c** *Wolbachia* density were measured. Each data point represents one cup of 150 mosquitoes (hatch and emergence) or one mosquito (*Wolbachia* density); 24–40 mosquitoes were sampled for each *Wolbachia* density group. Hatch rate data were analysed by ANOVA (not significant [ns]) and data are shown as the mean and standard error. Emergence rate and *Wolbachia* density data were analysed by generalised linear model and data are shown as medians with interquartile ranges

**Fig. 2:**
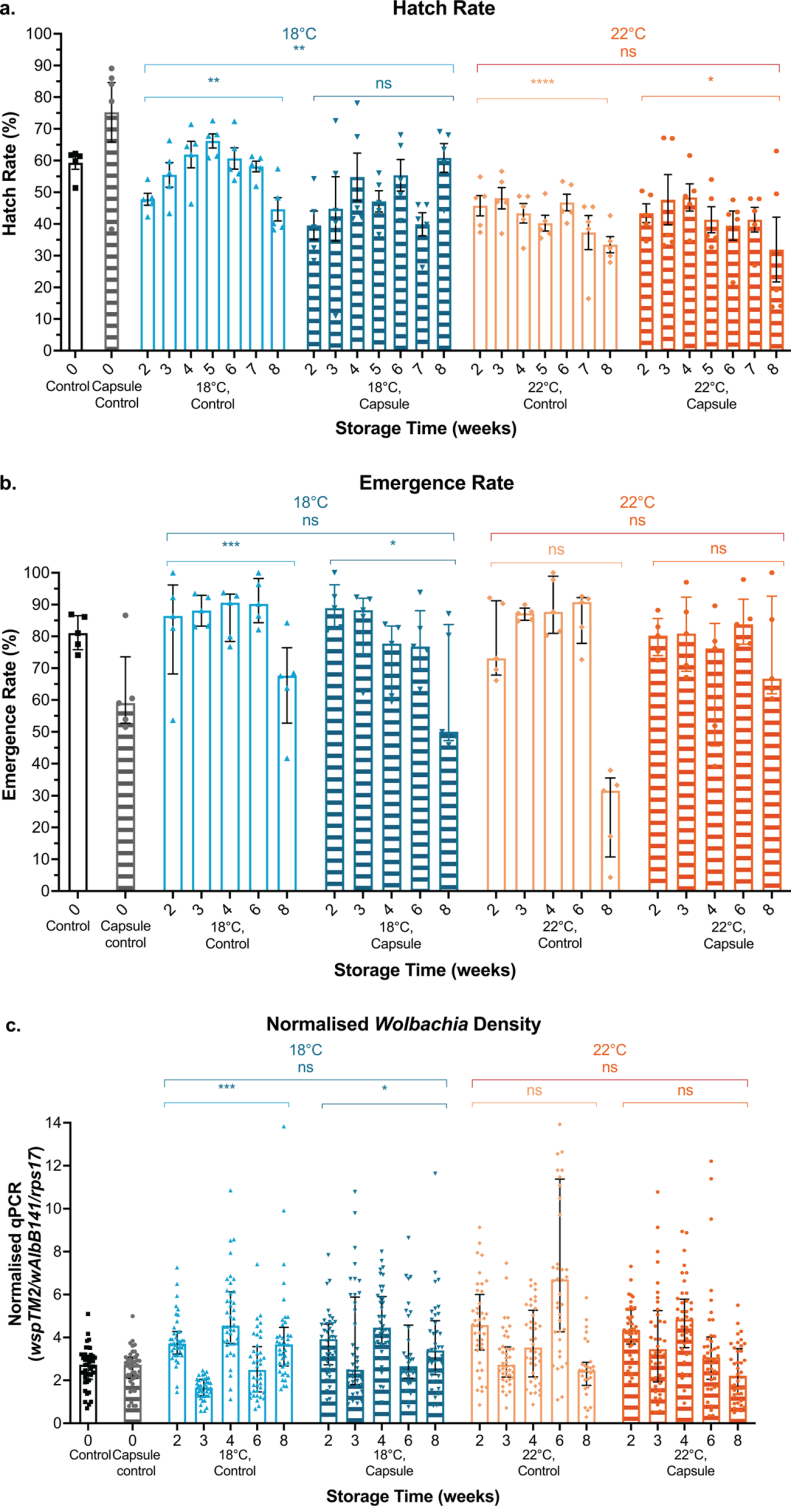
Encapsulating eggs for storage at 18 °C does not improve egg fitness compared to 22 °C. Eggs were packaged into water-soluble capsules with larval food or left on paper substrate as a control and stored at 18 °C or 22 °C (control) for 0, 2, 3, 4, 5, 6, 7 or 8 weeks. **a** Hatch rate **b** emergence rate and **c** *Wolbachia* densities were measured. Each data point represents one cup of 150 mosquitoes (hatch and emergence) or one mosquito (*Wolbachia* density); 24–40 mosquitoes were sampled for each *Wolbachia* density group. Hatch rate data were analysed by ANOVA followed by Tukey’s multiple comparison test (not significant [ns], *P* < 0.01**) and data are shown as the mean and standard error. The secondary significance bars compare hatch rate over time. Emergence rate and *Wolbachia* density data were analysed by generalised linear model and Kruskal-Wallis *H*-test (*P* < 0.05*, *P* < 0.001***) and data are shown as medians with interquartile ranges. Emergence rate secondary significance bars indicate change over time. *Wolbachia* density secondary significance bars compare week 8 to the corresponding week 0 control

**Fig. 3:**
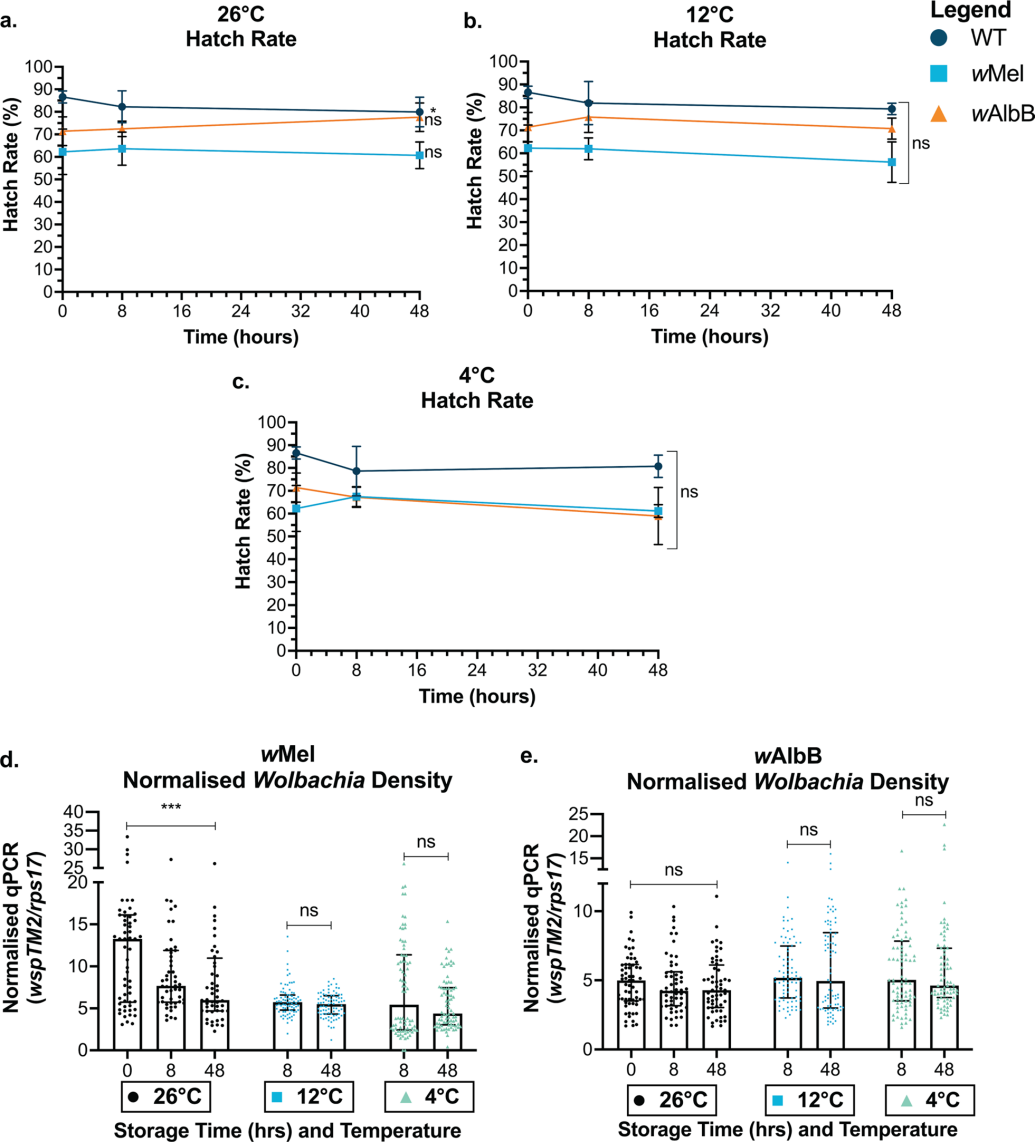
Impact of low egg storage temperatures on egg viability and adult *Wolbachia* density. WT, *w*Mel and *w*AlbB-infected eggs on paper substrate were stored at 26 °C, 12 °C and 4 °C for 0, 8 or 48 h. **a–c** Hatch rate and **d-e** *Wolbachia* density were measured. These data are representative of two combined experimental replicates. Each data point represents the average of three cups of 150–300 mosquitoes (hatch rate) or one mosquito (*Wolbachia* density); 80 mosquitoes were sampled for each *Wolbachia* density group. Hatch rate data were analysed by ANOVA followed by Tukey’s multiple comparison test (not significant [ns]) to compare changes in hatch rate over time within each group. Data are shown as the mean and standard deviation. *Wolbachia* density data were analysed by Kruskal-Wallis *H*-test and Wilcoxon signed rank test (*P* < 0.05*, *P* < 0.01**, *P* < 0.0001****) and data are shown as median with interquartile range

**Fig. 4:**
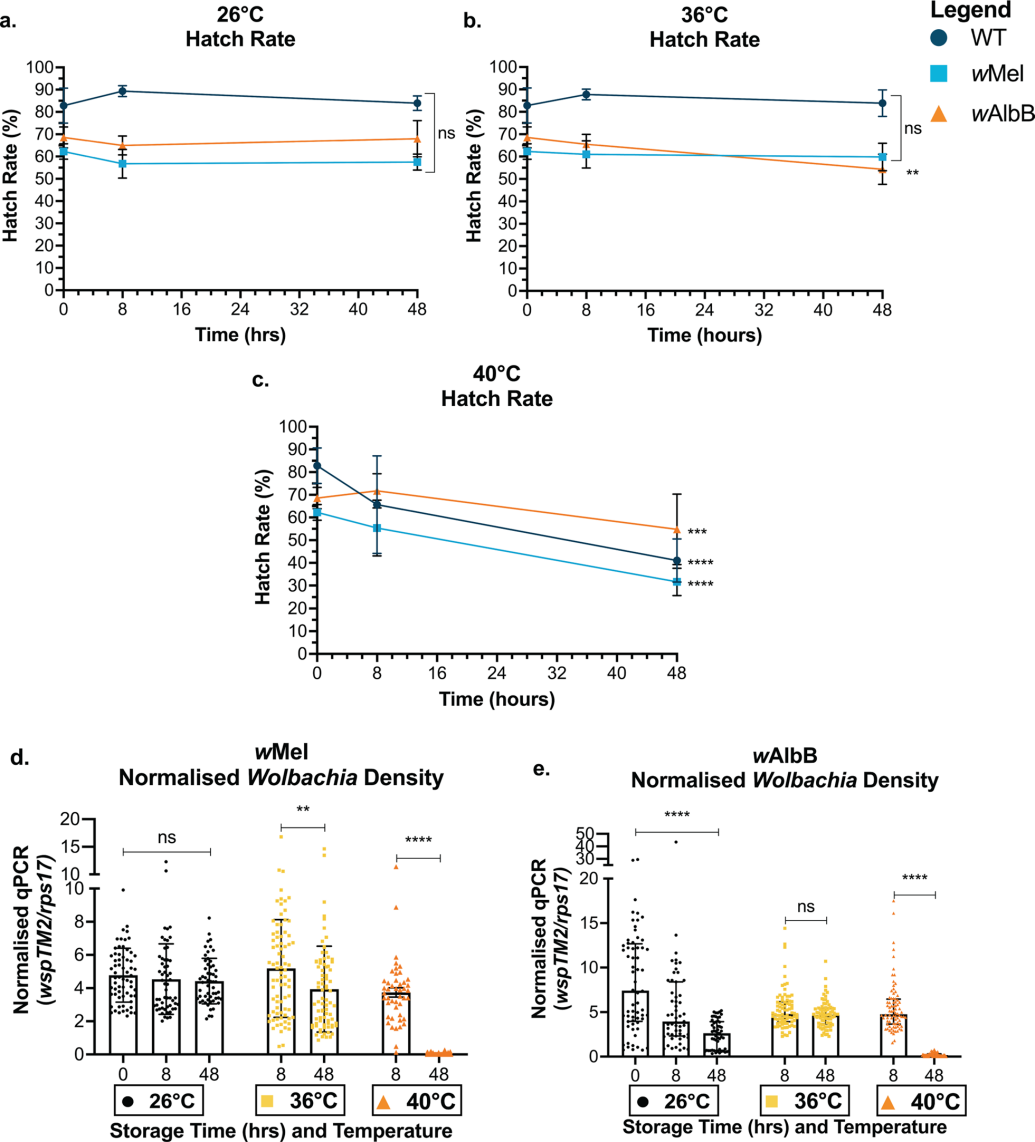
Impact of high egg storage temperatures on egg viability and adult *Wolbachia* density. Wild-type (WT), *w*Mel and *w*AlbB-infected eggs on paper substrate were stored at 26 °C, 36 °C and 40 °C for 0, 8 or 48 h. **a–c** Hatch rate and **d–e** *Wolbachia* density were measured. These data are representative of two combined experimental replicates. Each data point represents the average of three cups cup of 150–300 mosquitoes (hatch rate) or one mosquito (*Wolbachia* density); 80–115 mosquitoes were sampled for each *Wolbachia* density group. Hatch rate data were analysed by ANOVA followed by Tukey’s multiple comparison test (not significant [ns], *P* < 0.01**, *P* < 0.0001****) to compare changes in hatch rate over time within each group. Data are shown as the mean and standard deviation. *Wolbachia* density data were analysed by Kruskal-Wallis *H*-test and Wilcoxon signed rank test and data are shown as median with interquartile range

Data analysis was undertaken using R v 1.4.1717 and visualised using GraphPad Prism v 9.2. Normality was checked using the Shapiro-Wilk test and assumptions using diagnostic plots and residual simulation plots [56]. We performed a generalised linear model (GLM), Kruskal-Wallis *H*-test or Mann-Whitney U test (non-parametric data) [57–59]. Modelling was followed by ANOVAs to compare treatment effect (parametric data) [60]. If significant interactions were identified, we used Tukey’s *P*-value adjustment method for pairwise comparisons [61]. Two biological replicates were performed for each experiment, and we assessed whether replicate data were significantly different from each other to determine whether replicates were analysed separately or together. Fig. 1 and Additional file 1: Figure S1 and Fig. 2 and Additional file 1: Figure S2 were analysed independently and Figs. 3 and 4 are representative of two independent experiments analysed together including a replicate variable. Statistical outputs are provided in detail in Additional file 2: Dataset S1.

## Results

### Encapsulating eggs for storage at 22 °C does not exacerbate impacts on egg viability, adult emergence or Wolbachia density compared to controls

To assess the effect of encapsulating eggs on mosquito fitness, we stored WT, *w*Mel- and *w*AlbB-infected eggs inside capsules. We compared the effect of encapsulation, *Wolbachia* infection and 8-week storage time on quiescent egg longevity as well as the resulting adult emergence rates and *Wolbachia* density. When comparing each mosquito line hatched from eggs on paper substrate (control) to encapsulated eggs, hatch rate was not significantly impacted, but was influenced by storage time, with egg viability decreasing over time for all three mosquito lines (ANOVA; hatch rate: encapsulation, *F*_(1,148)_ = 9.1727, *P* = 0.7791; hatch rate: storage time, *F*_(1,148)_ = 31.9372, *P* < 0.0001****) (Fig. 1a). A repeat experiment showed a small but significant decrease in *w*Mel- and* w*AlbB-infected egg hatch rate when encapsulated (Additional file 1: Fig. S1a). Promisingly, emergence rates remained high, above an average of 75%, for all groups up to 8 weeks of storage and were not negatively impacted by encapsulation (GLM; P > 0.05 for all comparisons) (Fig. 1b). A repeat experiment showed similar trends, although a significant decrease in emergence at 8 weeks was observed in WT control and *w*AlbB-infected eggs regardless of encapsulation (Additional file 1: Fig. S1b). When analysing *Wolbachia* density of emerged adults, it was found that while density changed slightly over time (*w*AlbB generally increasing and *w*Mel decreasing or remaining consistent) (GLM; *Wolbachia* density: storage time, P = 0.0076**) encapsulation did not negatively impact *Wolbachia* density (GLM; *Wolbachia* density: encapsulation, *P* = 0.159) (Fig. 1c). The repeat experiment also demonstrated this (Additional file 1: Fig. S1c). Together, these experiments indicate that egg encapsulation does not exacerbate the negative impact of storage time on hatch rate, nor does it negatively impact on adult emergence or *Wolbachia* density in mosquitoes produced from encapsulated eggs stored for up to 8 weeks.

### Encapsulating eggs for storage at 18 °C does not improve egg fitness compared to 22 °C

We next assessed the impact of 18 °C storage temperature on encapsulated egg longevity and adult fitness, as results from Lau et al. [48] showed that storing *Wolbachia*-infected eggs at lower temperatures may extend egg longevity. This experiment focused on *w*Mel since this is the *Wolbachia* strain most widely used in field releases. Initially, we compared hatch rates of eggs from the paper substrate control or capsules stored at each temperature. At 18 °C, encapsulated egg hatch rate was significantly lower than in the controls (Tukey’s multiple comparison; 18 °C, control: capsule, *Z* = − 3.192 *P* = 0.0014**), while at 22 °C, the hatch rates of the control and encapsulated eggs were not significantly different to each other (Tukey’s multiple comparison; 22 °C, control: capsule, *Z* = − 1.118, P = 0.2634) (Fig. 2a). When considering the effect of temperature, hatch rates were slightly higher when stored at 18 °C compared to 22 °C for both control eggs and encapsulated eggs (Tukey’s multiple comparison; control, 18 °C: 22 °C, *Z* = 3.521 *P* = 0.0004***; capsule, 18 °C: 22 °C, *Z* = 2.124, *P* = 0.0337*). However, this was not found to be a repeatable difference (Additional file 1: Fig. S2). Combined, these results support that encapsulating eggs has no negative impact on egg viability compared to controls and suggest that decreasing storage temperature to 18 °C does not substantially impact egg viability.

Larvae were then reared to adulthood and emergence and *Wolbachia* density were assessed. Notably, we observed a reduction in emergence following 8 weeks of storage, which was not seen in Fig. 1b, but was observed in a repeat experiment (Additional file 1: Fig. S1b), perhaps due to batch variation in egg and food quality. Post hoc analysis revealed that the reduction in adult emergence observed over time was most significant for control eggs stored at 22 °C, but was not influenced by encapsulation or storage temperature (GLM; emergence: encapsulation, *P* = 0.6574; emergence: temperature, *P* = 0.2738) (Fig. 2b). Overall, emergence rate was not affected by egg encapsulation or storage at 18 °C compared to 22 °C. Adult *Wolbachia* density, while variable between groups, showed no clear trends of change with extended egg storage time (Fig. 2c). Most notably, no loss of *Wolbachia* was observed in any group (a critical concern for maintaining maternal transmission of *Wolbachia* in field releases), and encapsulation was not a source of variance for *Wolbachia* density (Kruskal-Wallis *H*-test; *Wolbachia* density: encapsulation, *H* = 1.164, *P* = 0.2806).

### Storage of *Ae. aegypti* eggs at 40 °C for 48 h induces lethality and *Wolbachia* loss

Mosquito eggs are transported from production facilities to release sites via air freight when local release sites do not have the capacity for large-scale production. When transporting eggs, ambient temperatures can reach extreme highs and lows, potentially impacting egg viability and *Wolbachia* density. Therefore, understanding the temperature range through which eggs remain viable, and *Wolbachia* is not negatively affected, is critical to ensure high quality control. To test this, we stored eggs on paper substrate at temperatures ranging from 4–40 °C and hatched after 8 or 48 h storage to assess egg viability. Low temperatures (4 °C and 12 °C) did not negatively impact egg viability of WT (Tukey’s multiple comparison, 0 h: 48 h; 12 °C, *Z* = − 2.051, *P* = 0.1002; 4 °C, *Z* = − 1.638, *P* = 0.2295), *w*Mel-infected (Tukey’s multiple comparison, 0 h: 48 h; 12 °C, *Z* = − 0.443, *P* = 0.8976; 4 °C, *Z* = 0.071, *P* = 0.9973) or *w*AlbB-infected eggs (Tukey’s multiple comparison, 0 h: 48 h; 12 °C, *Z* = − 0.299, *P* = 0.9519; 4 °C, *Z* = − 1.5 *P* = 0.2909) (Fig. 3a). Larvae were then reared at 26 °C and adults were sampled to measure *Wolbachia* density. *w*Mel density was negatively impacted by low temperatures (Kruskal-Wallis *H*-test; *w*Mel *Wolbachia* density: storage temperature, *H* = 17.614, *P* = 0.0002*** whereas *w*AlbB was not negatively impacted, but instead showed a slight increase in density (Kruskal-Wallis *H*-test; *w*AlbB *Wolbachia* density: storage temperature, *H* = 7.7577, *P* = 0.0208*) (Fig. 3d–e). There were two instances (out of 160 samples) of *w*Mel loss occurring when eggs were stored at 4 °C (Fig. 3d).

Next, we stored eggs at high temperatures of 36 °C and 40 °C. WT and *w*Mel-infected egg viability was maintained when eggs were exposed to 36 °C, while *w*AlbB-infected egg viability decreased slightly. All three lines had significantly decreased viability when stored at 40 °C for 48 h (Tukey’s pairwise comparison, 40 °C, 0 h: 48 h; WT, *Z* = − 7.36, *P* < 0.0001****; *w*Mel, *Z* = − 9.894, *P* < 0.0001****; *w*AlbB, *Z* = − 3.876, *P* = 0.0003***) (Fig. 4a–c). However, *w*AlbB-infected egg viability was inconsistent across replicate experiments. Both experimental replicates indicated a significant decrease in viability stored at 36 °C after 48 h, while in one experimental repeat, 40 °C had no significant impact on egg viability. Little to no impact was seen on *Wolbachia* density in adults that emerged from eggs stored at 36 °C (Wilcoxon signed-rank test, 36 °C *Wolbachia* density, 8 h: 48 h; *w*Mel, *Z* = − 3.0538, *P* = 0.0023**; *w*AlbB, *Z* = − 1.4402, *P* = 0.1498) (Fig. 4d). However, density was significantly decreased when eggs were stored at 40 °C for 48 h, where near complete *Wolbachia* loss was observed in the majority of both *w*Mel- and *w*AlbB-infected adults (Wilcoxon signed rank test, 40 °C *Wolbachia* density, 8 h: 48 h; *w*Mel, *Z* = − 8.2106, *P* < 0.0001****; *w*AlbB, *Z* = − 8.2106, *P* < 0.0001****) (Fig. 4e). These data demonstrate that if eggs are exposed to temperatures of 40 °C or above for 48 h they should be discarded as viability will be significantly decreased and adults that do emerge are unlikely to be *Wolbachia*-infected.

## Discussion

To date, *Wolbachia* has been successfully established in *Ae. aegypti* populations in cities across the globe to protect 10 million people from mosquito-borne diseases [50]. This remains a small proportion of the world’s population at risk of dengue, estimated to be 2.92–3.97 billion people [1]. As programmes such as those implementing *Wolbachia* introgression, gene drive or genetic modification, scale-up and work in new regions, they require a cost reductive and resource efficient method for mass mosquito releases. Release of mosquitoes at the egg stage is attractive as they can be produced off site and then shipped to release areas, removing the need for local mosquito-rearing facilities. Furthermore, they can be used to encourage community engagement by involving residents in the rear and release process [36]. This method overcomes financial and regulatory hurdles associated with establishing on-site facilities; however, maintaining egg quality and *Wolbachia* infection is essential for successful deployment [62]. Thus, egg and food capsules offer an opportunity to improve the scalability of egg releases. Our study tested the long-term storage of eggs inside of capsules as a method that could aid the mass distribution of eggs.

Promisingly, we found that encapsulating eggs has no negative impact on viability of WT, *w*Mel- or *w*AlbB-infected eggs. Over time, egg viability dropped in *Wolbachia*-infected and uninfected lines; however, encapsulation did not exacerbate this loss. There is extensive literature evidencing that *Wolbachia*-infected eggs lose viability faster than WT [40–48]. While it is still not clear why this occurs, it is important to know that encapsulation does not further impact egg viability over time. Emergence rate and adult *Wolbachia* density were also unaffected by encapsulation or storage time. Overall, regardless of *Wolbachia* infection status, encapsulated eggs were not more susceptible to reduced fitness.

Next, we tested whether reducing the storage temperature to 18 °C could improve encapsulated egg viability and adult fitness compared to 22 °C. While this is lower than the defined ideal range for WT *Aedes* eggs storage of between 20–26 °C and 70–85% RH from one study [63], others have shown that lower egg storage temperatures can extend egg longevity when compared with higher temperatures [48, 64]. We found that the viability of eggs infected with *w*Mel was not impacted by reducing storage temperature to 18 °C. The negative impact of egg storage increased with time, particularly in eggs stored at 22 °C, but this was irrespective of encapsulation. Consequentially, emergence rates were also reduced over time potentially due to an overabundance of food which can lead to poor water conditions unsuitable for aquatic mosquito health [65]. *Wolbachia* density was unaffected by encapsulation and time. While the impacts of 18 °C storage on egg viability were somewhat inconsistent here, future work could be done to determine whether an ideal temperature can be established for *Wolbachia*-infected *Ae. aegypti* egg longevity. Overall, encapsulating eggs and storing at 18–22 °C did not negatively impact mosquito fitness measures.

In field application of egg releases, the World Mosquito Program uses air freight to deliver eggs to release sites that cannot support mass production on site, which leaves eggs vulnerable to exposure to extreme temperatures. Currently, shipments aim to maintain temperatures between 15–25 °C. However, data loggers transported with the eggs indicate that temperatures can reach outside of this range, especially when being shipped to remote locations with shipment times of up to 5 days [62]. Gaining a detailed understanding of what conditions egg viability and *Wolbachia* density are vulnerable to will inform project sites of the potential impact on egg quality if egg stocks are exposed to extreme temperatures. We measured the impact of short-term (48-h) exposure of eggs to 4–40 °C on egg viability and resulting adult *Wolbachia* density. At 4 °C and 8 °C, egg viability and *w*AlbB density were unaffected. *w*Mel density decreased at 4 °C, but there were only two cases of *Wolbachia* loss out of the 160 samples tested. Previous studies have also demonstrated that *Ae. aegypti* eggs are tolerant to low temperatures, maintaining high viability when laid and stored at 16 °C [49, 67]. At high temperatures, egg viability and *Wolbachia* density were unaffected by exposure to 36 °C, but were significantly negatively affected when stored at 40 °C for 48 h. Loss of *Wolbachia* from egg stocks is detrimental because these eggs are no longer usable for *Wolbachia* introgression releases. In fact, releasing *Wolbachia*-free females would increase potential viral vectors within a population. All three lines behaved similarly, with the exception of *w*AlbB in one experimental repeat, which maintained viability at 40 °C despite reduced viability at 36 °C. Given the inconsistency of these results, it is unclear whether *w*AlbB eggs perform better at this higher temperature. *w*AlbB has been found to be relatively stable at high temperatures (26–37 °C) compared to *w*Mel [47, 68–70]. Ross et al. [69] found that while *w*AlbB was more temperature tolerant, egg viability decreased at a similar rate to *w*Mel-infected eggs when exposed to cycling temperatures for 1 week that reach a maximum of 38 °C or above. Adult *w*Mel density decreased after maximum egg storage temperatures of 36 °C, while *w*AlbB was maintained under all temperatures (egg viability was lost before a decrease in *w*AlbB was observed) [69]. Our data suggest that *w*AlbB is susceptible to drop out at acute high temperatures. Although *w*AlbB is more temperature tolerant, Lau et al. [48], showed that if eggs are stored at high temperatures (22–30 °C) for longer than 6 weeks, fertility of *w*AlbB-infected females derived from the eggs significantly decreases, while *w*Mel and WT fertility remains stable. In this study we did not observe a significant negative fitness effect of *w*AlbB. However, as our methods did not assess fertility, our results may underestimate the impacts of *w*AlbB on *Ae. aegypti*. Given this, care must be taken not to expose eggs to high temperatures when storing for long periods of time. While eggs were not encapsulated in these experiments, it is likely similar temperature limits would apply but further testing is required in case temperatures within capsules differ from ambient. These results provide insight into the impact of extreme temperature exposure on *Wolbachia*-infected *Ae. aegypti* eggs to ensure resources are not wasted on inviable egg stocks.

## Conclusions

In summary, our work has shown that encapsulating eggs with larval food and storing over an 8-week period does not negatively impact egg viability or resulting adult emergence and *Wolbachia* density compared to the control egg storage method. In addition, we established that egg viability and adult *Wolbachia* density are maintained well when exposed to 4–36 °C for 48 h, but both are significantly reduced when eggs are stored at 40 °C for > 8 h. Mass insect release biocontrol methods rely on the maintenance of insect fitness with *Wolbachia* introgression methods additionally requiring high *Wolbachia* infection prevalence. Capsule-based egg releases improve the ease and scale at which eggs and larval food can be consistently aliquoted and transported to the field. Compared to pupae or adult releases, capsules also provide a substantial logistical benefit for mass insect releases due to the reduced on-site resource requirements. Overall, this work improves our understanding of the factors that influence *Ae. aegypti* fitness and provides evidence for an improved egg release method that could aid large-scale application of *Wolbachia* introgression.

## Supplementary Information

**Additional file 1: Table S1.** qPCR primer and probe sequences. **Fig. S1**. Repeat experiment of data in Fig. 1: Encapsulating eggs for storage at 22 °C does not exacerbate impacts on egg viability, adult emergence or *Wolbachia* density compared to controls. **Fig. S2.** Repeat experiment of data in Fig. 2. Encapsulating eggs for storage at 18 °C does not improve egg fitness compared to 22 °C.

**Additional file 2: Dataset S1:** Statistical outputs.

## Data Availability

All data are provided within the text and Additional files.

## Abbreviations

*Ae*: *Aedes*
WT: Wild-type
DENV: Dengue virus
ZIKV: Zika virus
CHIKV: Chikungunya virus
RH: Relative humidity
RO: Reverse osmosis
*wsp*: *Wolbachia surface protein*
*RpS17*: *Ribosomal surface protein S17*
NE: Normalised expression
GLM: Generalised linear model
