# Supplementary material for: Enhancing the scalability of Wolbachia-based vector-borne disease management: time and temperature limits for storage and transport of Wolbachia-infected Aedes aegypti eggs for field releases
Source: Parasit Vectors. 2023 Mar 18;16:108. doi: 10.1186/s13071-023-05724-1 (PMC10024388; doi:10.1186/s13071-023-05724-1)
Supplement: Supplementary file 1 — Additional file 1: Table S1. qPCR primer and probe sequences. Fig. S1. Repeat experiment of data in Fig. 1: Encapsulating eggs for storage at 22 °C does not exacerbate impacts on egg viability, adult emergence or Wolbachia density compared to controls. Fig. S2. Repeat experiment of data in Fig. 2. Encapsulating eggs for storage at 18 °C does not improve egg fitness compared to 22 °C. [file 13071_2023_5724_MOESM1_ESM.docx]

**Table S1.** qPCR primer and probe sequences. Reference gene *Ribosomal Protein S17 (RpS17)* [51] and target genes *Wolbachia surface protein (wsp)* [52] and the ankyrin repeat domain-containing protein (DEJ70_01140) in *w*AlbB (*wAlbB141*).

| Primer name | Sequence (5’-3’) |
| --- | --- |
| wspTM-LC640 | LC640-TCCTTTGGAACCCGCTGTGAATGA-IowaBlack |
| wspTM-2F | CATTGGTGTTGGTGTTGGTG |
| wspTM-2R | ACACCAGCTTTTACTTGACCAG |
| Rps17_TaqM_FW | TCCGTGGTATCTCCATCAAGCT |
| Rps17_TaqM_RV | CACTTCCGGCACGTAGTTGTC |
| Rps17_TaqM_Probe | FAM-CAGGAGGAGGAACGTGAGCGCAG-BHQ1 |
| wAlbB141 (F) | AGTAGTGCAGCGAGTCT |
| wAlbB141 (R) | TGGAGGAAGAGTTCACTGTGC |
| wAlbB141 (FAM) | FAM-ZEN-AATTATCCCCTACCAAAGCAATTAAGATAGAAT-IowaBlack |


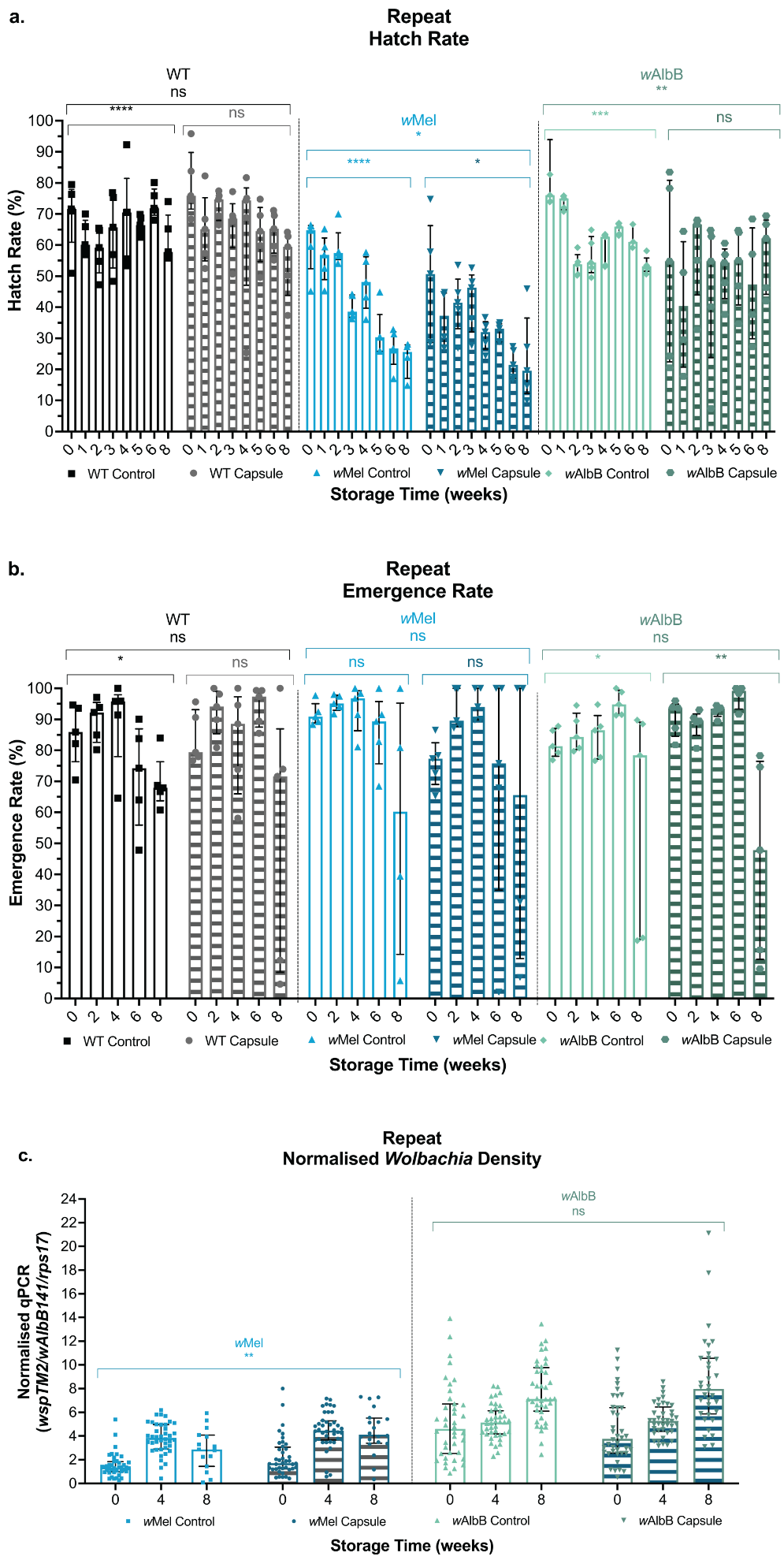
**Fig. S1.** Repeat experiment of data in Fig. 1: Encapsulating eggs for storage at 22°C does not exacerbate impacts on egg viability, adult emergence or *Wolbachia* density compared to controls. WT, *w*Mel- and *w*AlbB- infected eggs were packaged into water-soluble capsules with larval food or left on paper substrate as a control and stored at 22°C for 1, 2, 3, 4, 5, 6 or 8 weeks. **(a)** Hatch rate **(b)** emergence rate and **(c)** *Wolbachia* density were measured. Each data point represents one cup of 150 mosquitoes (hatch and emergence) or one mosquito (*Wolbachia* density). 24-40 mosquitoes were sampled for each *Wolbachia* density group. Data were analysed by Kruskal-Wallis H-test (not significant (ns), P<0.05*, P<0.01**, P<0.001***, P<0.0001****) and data are shown as medians with interquartile ranges. The secondary significance bars indicate change over time.


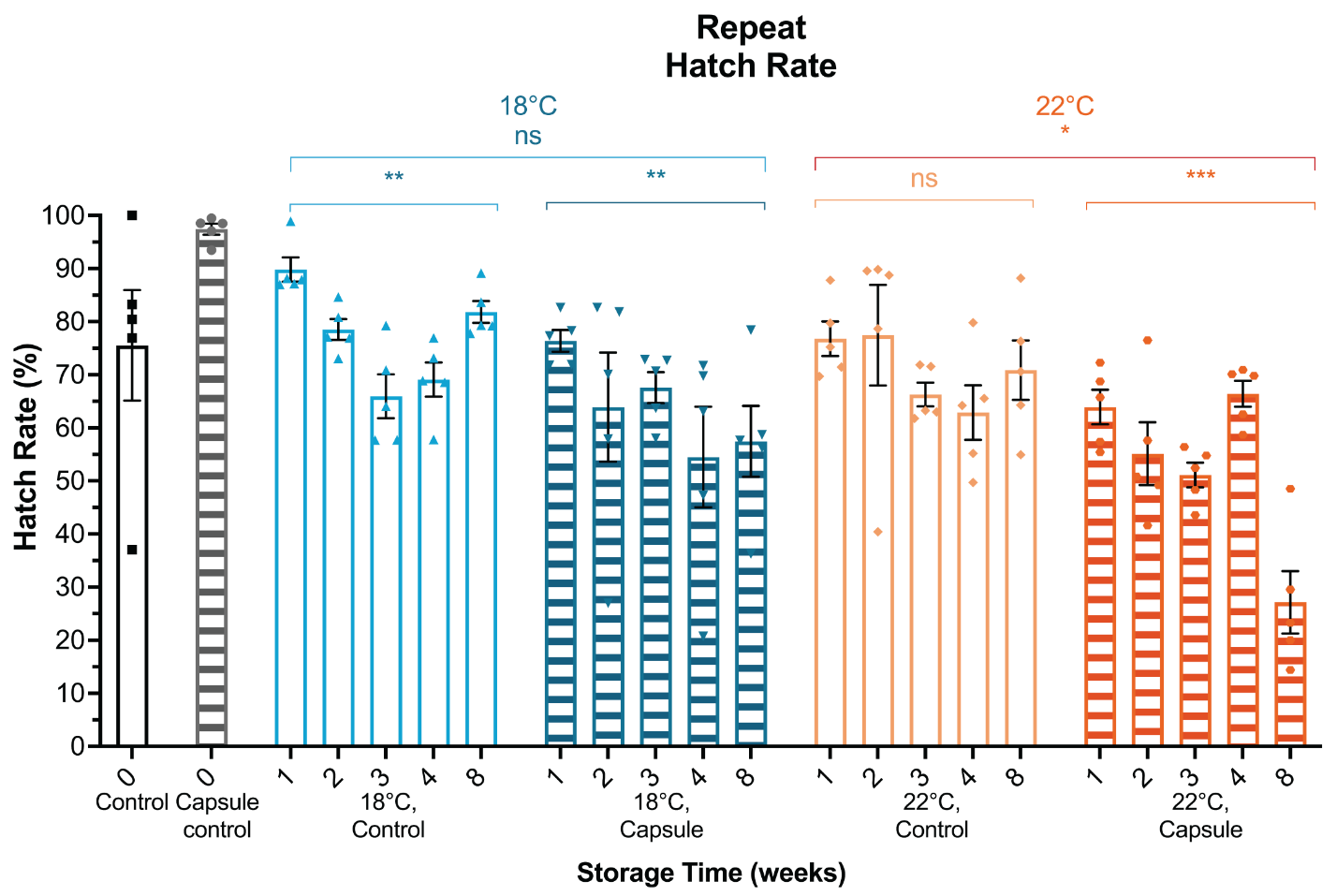
**Fig. S2.** Repeat experiment of data in Fig. 2: Encapsulating eggs for storage at 18°C does not improve egg fitness compared to 22°C. Eggs were packaged into water-soluble capsules with larval food or left on paper substrate as a control and stored at 18°C or 22°C for 0, 1, 2, 3, 4 or 8 weeks and hatch rate was measured at each time point. Each data point represents one cup of 150 mosquitoes. Data was analysed by Kruskal-Wallis H-test (not significant (ns)), and data is shown as median with interquartile range. Hatch rates were not different when stored at 18°C compared to 22°C for control eggs, however, were slightly higher when stored at 18°C compared to 22°C for encapsulated eggs (Kruskal-Wallis H-test; control, 18°C : 22°C, P=0.1579; capsule, 18°C : 22°C, P=0.0315*).
